# Genetic diversity and floral width variation in introduced and native populations of a long-lived woody perennial

**DOI:** 10.1093/aobpla/plu087

**Published:** 2014-12-19

**Authors:** Jane C. Stout, Karl J. Duffy, Paul A. Egan, Maeve Harbourne, Trevor R. Hodkinson

**Affiliations:** 1 School of Natural Sciences and Trinity Centre for Biodiversity Research, Trinity College Dublin, Dublin 2, Ireland; 2 School of Life Sciences, University of KwaZulu-Natal, Private Bag X01, Scottsville, Pietermaritzburg 3209, South Africa

**Keywords:** AFLP, corolla tube, floral morphology, invasive plants, microsatellites, population differentiation, SSR.

## Abstract

Populations of introduced species in their new environments are expected to differ from native populations, due to processes such as genetic drift, founder effects and local adaptation, which can often result in rapid phenotypic change. Such processes can also lead to changes in the genetic structure of these populations. This study investigated the populations of *Rhododendron ponticum* in its introduced range in Ireland, where it is severely invasive, to determine both genetic and flower width diversity and differentiation. We compared six introduced Irish populations with two populations from *R. ponticum*'s native range in Spain, using amplified fragment length polymorphism and simple sequence repeat genetic markers. We measured flower width, a trait that may affect pollinator visitation, from four Irish and four Spanish populations by measuring both the width at the corolla tip and tube base (nectar holder width). With both genetic markers, populations were differentiated between Ireland and Spain and from each other in both countries. However, populations displayed low genetic diversity (mean Nei's genetic diversity = 0.22), with the largest proportion (76–93 %) of genetic variation contained within, rather than between, populations. Although corolla width was highly variable between individuals within populations, tube width was significantly wider (>0.5 mm) in introduced, compared with native, populations. Our results show that the same species can have genetically distinct populations in both invasive and native regions, and that differences in floral width may occur, possibly in response to ecological sorting processes or local adaptation to pollinator communities.

## Abstract (precis)

We demonstrate that invasive populations of *Rhododendron ponticum* in Ireland are genetically distinct from ancestral populations in Spain and produce flowers which have wider floral tubes. Although Irish populations are spreading and Spanish ones are declining, we found low genetic diversity among individual plants within populations in both regions, and limited between-population gene flow. Wider floral tubes may have evolved in response to novel pollinators in Ireland. Few studies examine invasive species in both their introduced and native habitats, but this approach is needed to understand invasive species evolution and ecology.

## Introduction

When a species is introduced outside its native range as a result of human activity, genetic diversity and morphological variability within and between populations can vary. This may be a result of the phenotypic plasticity of the species (Chun 2011; Godoy *et al.* 2011), propagule pressure (the number of individuals released and the number of release events; Lockwood *et al.* 2005, but see Nuñez *et al.* 2011) and post-introduction evolutionary processes like inbreeding, drift, hybridization and response to novel selection pressures (Lee 2002; Prentis *et al.* 2008). Introduced populations may be expected to display low genetic diversity due to founder effects from a limited number of initially introduced individuals, with associated negative fitness consequences (Ellstrand and Elam 1993; Young *et al.* 1996), although this is not always the case. Populations of two highly successful introduced plants, *Fallopia japonica* (Japanese Knotweed) and *Eichhornia crassipes* (water hyacinth), have been shown to have very low genetic diversity (Hollingsworth and Bailey 2000; Ren *et al.* 2005).

Post-introduction changes in genetic and morphological variability in plant species may also be related to the life form and breeding system. For example, pollinator limitation, as a result of separation of an introduced plant from its native pollinators, may exert selection pressure on self-compatible populations to evolve from self-incompatible ancestors to ensure seed set (Petanidou *et al.* 2012). This is less likely in perennial species, which have more opportunities for sexual reproduction and the possibility of vegetative spread, and they may retain high genetic diversity post-introduction (Hamrick and Godt 1996). Another possibility is that rather than selection for self-compatibility, introduced plants may be more attractive to resident native pollinators. For example, this could be as a result of the production of larger flowers, which can be more attractive to foraging insects (Eckhart 1993; Conner and Rush 1996). Furthermore, specialized floral characteristics that restrict access to floral rewards, such as narrow or long floral tubes (Suzuki 1994; Stang *et al.* 2006), may be selected against and plants that have more easily accessible rewards may receive more frequent pollinator visitation in their new environment (Armbruster and Baldwin 1998). In self-incompatible *Ipomopsis aggregata*, floral width is under strong selective pressure (Campbell *et al.* 1996) because wider flowers allow increased bill insertion by hummingbird pollinators and a greater proportion of pollen removal. Stang *et al.* (2006) found that in a Spanish floral community there were more visitors to flowers with wide nectar holders. Hence, floral width, particularly nectar holder width, may be an important floral trait determining attractiveness to floral visitors and potential pollinators.

Here we investigate population genetic diversity and floral width variation of *Rhododenron ponticum* (Ericaceae) in expanding introduced populations and compare these with declining native populations. This long-lived woody species was once widely distributed throughout Europe (Cross 1975; Chamberlain *et al.* 1996; Milne and Abbott 2000), but now is primarily found in northern Turkey, the Caucasian states, Lebanon, southern Bulgaria and the Iberian Peninsula (Cross 1975, 1981; Colak *et al.* 1998; Rotherham 2001; Mejías *et al.* 2002). Iberian populations are small and confined to three isolated areas, the largest of which is in the Aljibe Mountains in southern Spain (Castroviejo *et al.* 1993; Mejías *et al.* 2002), where it is classified as endangered (Blanca *et al.* 2000) under IUCN red list criteria (IUCN 1994). In this region it is known from ∼20 populations, which, although not undergoing rapid decline (Mejías *et al.* 2007), suffer from very low recruitment, and thus it is considered a vulnerable species in the area (VU: Cabezudo *et al.* 2005). *Rhododenron ponticum* was introduced as an ornamental plant into Britain and Ireland in the late 18th century (Milne and Abbott 2000; Dehnen-Schmutz and Williamson 2006) and now forms large invasive populations, which are spreading into, and having negative impacts on, native ecosystems on both islands (Cross 1975, 1981; Colak *et al.* 1998). Repeated introductions into many locations over time have created intense propagule pressure (Stephenson *et al.* 2006). Molecular analysis of chloroplast and nuclear ribosomal DNA indicated that British and Irish populations are predominantly derived from Spanish populations, and that hybridisation with North American species (*Rhododendron catawbiense* and *R. maximum*) occurred after *R. ponticum* was introduced in Britain (Milne and Abbott 2000). This was thought to have contributed to the competitive success of populations of *R. ponticum* in Ireland (Erfmeier and Bruelheide 2005; Erfmeier and Bruelheide 2010), where it is particularly successful in the Atlantic climate of the west coast (Cross 1981). However, analyses using amplified fragment length polymorphism (AFLP) markers showed that hybridization is unlikely to have contributed to invasiveness in Irish populations (Erfmeier *et al.* 2011). Relatively low genetic diversity was found in both Irish and Spanish populations, compared with Georgian ones, with weak genetic differentiation among populations within the three countries (Erfmeier and Bruelheide 2011). Studies of growth traits and life history have found that Irish populations had higher rates of annual growth and seedling recruitment (Erfmeier and Bruelheide 2004), and suggest a genetic basis for these traits (Erfmeier and Bruelheide 2005). A previous study comparing the pollination ecology of Irish and Spanish populations has shown that a range of generalist pollinator species visit *R. ponticum* flowers in both ranges, although visitor communities are dominated by different species in Ireland versus Spain, and that a greater volume of nectar is produced in plants from introduced populations (Stout *et al.* 2006). This may indicate that these populations contain individuals with wider flowers that hold greater quantities of nectar. However, no previous studies have investigated floral traits (e.g. nectar holder width) in introduced populations of *R. ponticum*, which may be important given that this species has a mixed mating system, primarily relying on animal-mediated outcrossing to produce seeds for invasion (Mejías *et al.* 2002; Stout 2007*a*). Selective pressures or ecological sorting in the introduced range could have resulted in larger, more open flowers to enhance attractiveness to resident generalist pollinators.

We examined genetic and floral morphological diversity within and between populations of *R. ponticum* in Ireland, and compared them with native populations in the ancestral range in the Aljibe Mountains in Spain. The objectives were to: (i) quantify genetic diversity both within and among introduced and native populations; (ii) determine genetic differentiation among introduced and ancestral populations; and (iii) quantify floral width (corolla width and tube width) in the introduced and native range. Specifically, we tested the hypothesis that there is genetic and floral width differentiation among populations in Ireland, and between Irish and Spanish populations.

## Methods

### Leaf sampling and DNA extraction

Sampling for genetic analysis was carried out in six Irish populations (Table 1), chosen to cover the geographic range of *R. ponticum* within the country, including the west coast (County Galway), the south-west (County Kerry) and the east (County Dublin). Irish populations were relatively large (>100 adult plants, Table 1). In addition, two Spanish populations were sampled within the Parque Natural Los Alcornocales (∼5 km inland from the Strait of Gibraltar). These populations were sampled in 2002; they were the largest populations in the Los Alcornacales region, but were still comparatively small (18 and 27 adult plants per population). All of the Spanish populations occur within an ∼50 × 30 km area, and are mostly confined to the Aljibe Mountains, where they are restricted to riparian forest habitats (Mejías *et al.* 2007). Nine to 12 individual plants within both introduced and native populations were randomly selected from each population (Table 1). To avoid sampling clones, distinct individuals, separated by >5 m, were selected. We used this sampling procedure as previous work has shown that vast majority of pollinator visits occur within-plant and that the majority of seeds land close to maternal plants (Stephenson *et al.* 2007; Stout 2007*b*). In addition, this sampling procedure ensured that replicate samples were taken in the native range to compare with invasive populations.

**Table 1.** *Rhododendron ponticum* populations used for genetic analysis and genetic diversity estimates within populations using (i) AFLP markers and (ii) SSR markers. Size, approximate number of mature, flowering plants in a population; *N*, number of individuals analysed; Tb, total number of bands; Pb, number of private bands; *P*, percentage of polymorphic loci at the 5 % level; *H*_j_, Nei's genetic diversity; *N*_a_, observed allele number; *N*_e_, effective allele number; *H*_O_, observed heterozygosity; *H*_E_, expected heterozygosity; *H*, average heterozygosity.

Region	Population	Position	Size	(i) AFLP						(ii) SSR				
				*N*	Tb	Pb	*P*	*H*_j_	*N*	*N*_a_	*N*_e_	*H*_O_	*H*_E_	*H*
Ireland	Howth	53.377N 6.07W	∼150	10	277	7	68.9	0.234	10	3.25	2.64	1.000	0.591	0.48
	Glencullen	53.23N 6.272W	∼150	9	268	13	66.7	0.226	10	2.50	2.30	1.000	0.547	0.48
	Gortderraree	51.988N 9.558W	>1000	10	254	6	63.2	0.218	10	3.00	2.86	0.750	0.465	0.49
	Gortracussane	52.006N 9.54W	>1000	10	239	2	59.5	0.211	11	3.75	2.92	0.750	0.494	0.48
	Recess	54.467N 0.739W	∼100	10	274	5	68.2	0.227	10	3.25	2.89	1.000	0.672	0.48
	Kylemore	53.561N 9.866W	>1000	10	264	4	65.7	0.204	10	3.00	2.57	0.750	0.450	0.49
Spain	El Palancar	36.082N 5.543W	18	10	274	14	68.2	0.225	10	2.00	2.00	0.500	0.250	0.53
	Las Corzas	36.111N 5.528W	27	10	278	14	69.2	0.225	12	3.50	2.44	0.500	0.289	0.53

Leaf material was collected and stored in silica gel (Chase and Hills 1991). DNA was extracted from ∼0.1 g of dried material using a modified 2× hexadecyltryltrimethyl-ammonium bromide procedure (Doyle and Doyle 1987; Hodkinson *et al.* 2007), and was purified with JetQuick columns (GENOMED Gmbh) according to the manufacturer's protocol. Two polymerase chain reaction (PCR)-based methods were employed to assess genetic diversity: AFLPs (Vos *et al.* 1995) and nuclear microsatellites—simple-sequence repeats (SSRs).

### AFLP protocol

Sampled DNA was restricted with the endonucleases EcoRI and MseI and ligated to appropriate double-stranded adapters according to the manufacturer's protocols. Amplified fragment length polymorphism analysis was performed according to the AFLP plant mapping protocol of Applied Biosystems, Inc. Two steps of amplification followed: a pre-selective amplification using primer pairs with one selective base was followed by a selective amplification to further reduce the number of fragments. For the second amplification, the following three selective primer pairs were selected sequentially: EcoRI-ACA/MseI-CAG, EcoRI-AAG/MseI-CTC and EcoRI-AGC/MseI-CAG. The products were sized using an Applied Biosystems 310 Genetic Analyzer with GeneScan version 3.1 and Genotyper version 3.7 software. Amplified fragment length polymorphism profiles were manually scored with the presence of each peak recorded as ‘1’ and the absence of a peak as ‘0’. Only peaks ranging from 50 to 500 bp were scored. A peak was scored as present if it was separated by at least 1 bp and has a relatively high peak height threshold (Meudt and Clarke 2007). In order to reduce genotyping error, AFLP profiles were scored at least twice by individuals with no knowledge of the origin of plant material.

### SSR protocol

No SSR markers have been published for *R. ponticum*, and so nuclear SSR amplification of seven polymorphic loci isolated from *R. metternichii* var. *hondoense* was screened according to the methods described in Naito *et al.* (1998), of which four were informative for *R. ponticum* (RM3D2, RM2D2, RM9D6 and RM2D5). Polymerase chain reaction amplification followed (Naito *et al.* 1998), and the amplicons were sized on an Applied Biosystems 310 Genetic Analyzer with GeneScan version 3.1 and Genotyper version 3.7 software.

### Floral width

In addition to quantifying genetic variation in native and invasive populations of *R. ponticum*, in 2011 we quantified floral width in representative plants in both regions to test whether nectar holder width varied between populations and the two regions. To estimate floral width, two measurements were made on each flower in the field using dial callipers (Moore and Wright, CDP150M), with a precision of 0.01 mm: (i) the width of the corolla at the widest point between the upper wing petals and (ii) the width of the corolla tube at the base. These traits were measured as they represent the extent to which *R. ponticum* flowers are open to insect pollinators in order to access nectar rewards. Due to logistical constraints, and the fact that these data were collected separately from the leaf material for the population genetic study, only relatively few measurements were taken per population and in only one of the populations (El Palancar, Spain) sampled for genetic analysis. Measurements were made in four Irish and four Spanish populations (Table 2). From each population, five completely open flowers (third floral phase, i.e. with corolla wide open, stigma receptive and protruding beyond anthers; Mejías *et al.* 2002) from each of five individual plants were randomly selected for measurement.

**Table 2.** *Rhododendron ponticum* populations used for flower morphology measurements.

Region	Population	Position	Size	Elevation (m)	Habitat type
Ireland	Crossover	52.894N 6.400W	75	165	Riparian woodland, *Quercus petraea* dominant, with *Betula pendula*
	Dunran	53.060N 6.102W	125	156	Mixed forest plantation, mainly *Pinus contorta* with *Q. petraea* in patches
	Tropperstown	53.017N 6.274W	50	185	Open forest, *Q. petraea*, *Fraxinus excelsior* and *B. pendula* dominant
	Shankhill	53.192N 6.427W	225	281	Mixed forest plantation, *Fagus sylvatica* and *F. excelsior*, some *P. contorta*
Spain	El Palancar	36.081N 5.543W	50	495	Stream gulley, patchy *Q. suber* forest on the edge of grazed grassland
	Llanos del Juncal	36.105N 5.540W	125	747	Cloud forest, *Q. canariensis* with *Crataegus monogyna* and *Ilex perado*
	Garganta de Puerto Oscuro	36.518N 5.632W	100	605	Stream valley, mixed forest cover of *Q. canariensis* with *Q. suber* patches
	Garganta del Aljibe	36.538N 5.635W	75	469	Stream valley, *Q. canariensis* dominant, *Arbutus unedo* common

### Data analysis

#### Population genetic diversity

For the AFLP data set, genetic diversity estimates were calculated with AFLPsurv V.1.0 (Vekemans *et al.* 2002). To estimate allelic frequencies, the Bayesian method with a non-uniform prior distribution of allele frequencies (Zhivotovsky 1999) was used. Due to the mixed mating system of the species (Stout 2007*a*), we assumed some deviation (*F*_IS_ = 0.1) from the Hardy–Weinberg equilibrium. Statistics of gene diversity were calculated according to Lynch and Milligan (1994). For each population, we calculated the proportion of polymorphic loci (*P*) and Nei's gene diversity (*H*_j_). For the SSR data set, GenAlEx 6.2 (Peakall and Smouse 2006) was used to test for departures from the Hardy–Weinberg equilibrium. Observed heterozygosity (*H*_O_) and Nei's expected heterozygosity (*H*_E_) were calculated with GenAlEx 6.2, and the average heterozygosity was calculated with PopGene 1.32 (Yeh *et al.* 2000).

#### Population genetic structure

Euclidean pairwise genetic distances were calculated in GenAlEx 6.2, which allows a common pathway for subsequent statistical analysis for both dominant AFLP markers and codominant SSR markers (Maguire *et al.* 2002). For both data sets, genetic distances were calculated using Eq. (1),

(1) E=n[1−(2nxy/2n)]

where *n* is the total number of polymorphic bands and 2*nxy* is the number of markers shared by two individuals (Peakall *et al.* 1995; Maguire *et al.* 2002). Total genetic diversity was partitioned among groups of populations, among populations within groups and within populations using a hierarchical analysis of molecular variance (AMOVA) in GenAlEx 6.2. Genetic structure was tested with AMOVA on the genetic distance matrix (9999 permutations) produced for both sets of markers (Weir 1996). Analysis of molecular variance output nomenclature follows that of Excoffier *et al.* (1992) in that variation was summarized both as the proportion of the total variance and as *φ*-statistics (*F*_ST_ analogues). Pairwise genetic distances among populations and their level of significance for both the AFLP and SSR markers were also obtained from the AMOVA (9999 permutations). In addition, a non-hierarchical AMOVA was performed to test population differentiation in Ireland and Spain separately.

Unweighted pair group method with arithmetic mean cluster analysis (UPGMA) was performed in PopGene 1.32 using Nei's genetic distance (Nei 1972) to analyse the patterns of population-level genetic distances across all populations for both the AFLP and SSR data sets. A Mantel test was used to compare pairwise genetic differences from the AFLP and SSR data.

#### Floral width

Corolla width and tube width were compared between regions (Ireland and Spain), among populations within regions and among plants within populations, using hierarchical (nested) ANOVA (with ‘region’, Ireland or Spain, as a fixed factor, ‘population’ nested within the region as a random factor and ‘plant’ nested within the population as a random factor; *n* = 5). Analyses were conducted using GMAV5 for Windows (University of Sydney, Australia). Data were tested for heterogeneity of variances using Cochran's test prior to analysis (*P* = 0.0976 and 0.0978 for corolla and tube width data, respectively) and were not transformed. *Post-hoc* Student–Newman–Keuls (SNK) tests were used to determine which means differed from each other (using the standard threshold of significance *α* = 0.05).

## Results

### AFLP markers

**Figure 1.**
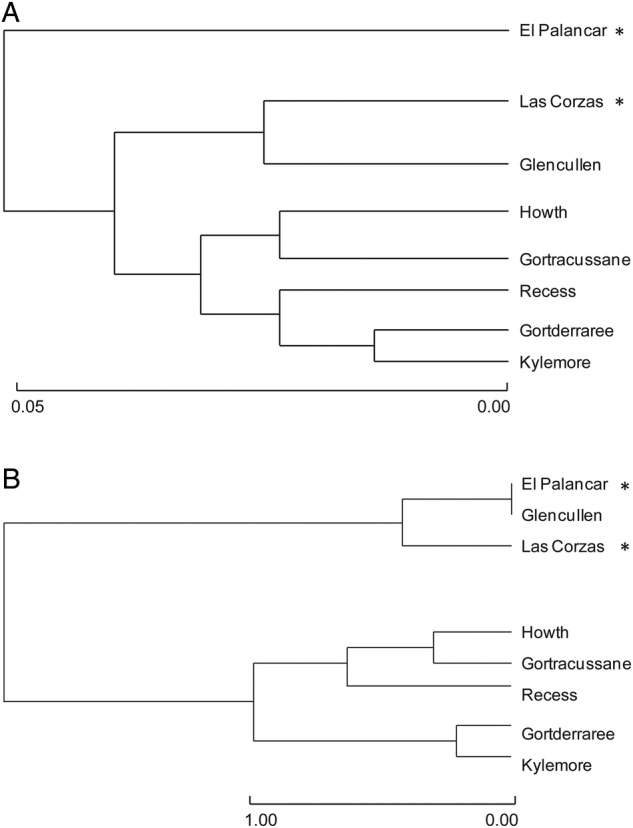
Rooted UPGMA tree depicting relationships between the populations investigated based on: (A) the AFLP data set and (B) the SSR data set using Nei's genetic distance (Nei 1972). Asterisks indicate native Spanish populations and scale bars represent genetic distance.

A total of 402 reliable peaks were produced from the three AFLP primer combinations: 132 EcoRI-ACA/MseI-CAG, 144 EcoRI-AGC/MseI-CAG and 126 EcoRI-AAG/MseI-CTC among the 79 *R. ponticum* individuals surveyed. Overall, 95.3 % of the loci were polymorphic. Genetic diversity (*H*_j_) was similar within both Irish and Spanish populations (range 0.204–0.234 over all populations) and more than half (59–69 %) of loci within populations were found to be polymorphic (Table 1). Analysis of molecular variance revealed that 93 % of the variance was found among individuals within populations (Table 3a). Significant (*P* < 0.001) but low genetic differentiation was recorded among populations relative to the total (*φ*_PT_ = 0.070), among populations within regions (Ireland and Spain) (*φ*_PR_ = 0.044, *P* < 0.001) and among regions (*φ*_RT_ = 0.028, *P* = 0.005) (Table 3a). Pairwise *φ*_PT_ values between populations were variable, ranging from <0.001 to 0.133 (Table 4a). A non-hierarchical AMOVA (not shown) revealed less, although significant, differentiation among Irish populations (*φ*_PT_ = 0.037; *P* = 0.001) than among Spanish populations (*φ*_PT_ = 0.073; *P* = 0.01). When individuals were grouped into populations, the UPGMA separated the Spanish population, El Palancar from other populations, and grouped the Las Corzas population with the Irish populations (Fig. 1A).

**Table 3.** Analysis of molecular variance based on: (a) 402 AFLP loci and (b) four nuclear SSR loci. *φ*_RT_ is the correlation of individuals from the same region (Spain or Ireland) relative to that of the total; *φ*_PR_ is the correlation between individuals within a population relative to that of individuals within the same region; *φ*_PT_ is the correlation between individuals within a population relative to that of individuals of the total (Peakall *et al.* 1995).

(a)					
Source	Df	SS	MS	Variation	%
Between regions	1	100.333	100.333	1.279	3
Among populations	6	371.499	61.916	1.947	4
Within populations	71	3033.128	42.720	42.720	93
	*φ* value	*P* value			
*φ*_RT_	0.028	0.004			
*φ*_PR_	0.044	<0.001			
*φ*_PT_	0.070	<0.001			
**(b)**					
**Source**	**Df**	**SS**	**MS**	**Variation**	**%**
Between regions	1	16.066	16.066	0.333	13
Among populations	6	30.702	5.117	0.304	12
Within populations	75	148.823	1.984	1.984	76
	*φ* value	*P* value			
*φ*_RT_	0.127	<0.001			
*φ*_PR_	0.133	<0.001			
*φ*_PT_	0.243	<0.001

**Table 4.** Genetic distances (Nei 1972) based on the (a) AFLP data set and (b) SSR data set. Values and levels of significance are given in the lower left and upper right of triangle, respectively. Significances are based on random permutations (9999). *P< 0.05 , **P < 0.01 ,***P < 0.001.

(a)								
Spain		Ireland						
El Palancar	Las Corzas	Howth	Glencullen	Gortderraree	Gortracussane	Recess	Kylemore	*Spain*
	**	*	***	**	***	**	***	El Palancar
0.073		ns	ns	*	**	ns	**	Las Corzas
								*Ireland*
0.039	0.036		ns	ns	ns	ns	*	Howth
0.093	0.041	0.028		ns	*	ns	*	Glencullen
0.072	0.071	<0.001	0.022		*	ns	**	Gortderraree
0.104	0.090	0.026	0.041	0.039		ns	*	Gortracussane
0.092	0.034	0.028	0.041	0.040	0.040		ns	Recess
0.133	0.087	0.051	0.049	0.086	0.053	0.020		Kylemore
**(b)**								
Spain		Ireland						
El Palancar	Las Corzas	Howth	Glencullen	Gortderraree	Gortracussane	Recess	Kylemore	*Spain*
	ns	***	**	***	**	***	***	El Palancar
0.104		***	**	**	**	***	***	Las Corzas
								*Ireland*
0.376	0.292		*	**	ns	ns	***	Howth
0.176	0.196	0.133		**	ns	**	**	Glencullen
0.212	0.147	0.236	0.134		ns	**	*	Gortderraree
0.184	0.140	0.049	0.069	0.030		ns	*	Gortracussane
0.387	0.305	0.062	0.174	0.131	<0.001		**	Recess
0.427	0.323	0.298	0.252	0.094	0.110	0.125		Kylemore

### SSR markers

A total of 29 alleles were detected within the 83 *R. ponticum* individuals and across the four nuclear SSR loci examined (RM3D2 = 6, RM2D2 = 9, RM9D6 = 8, RM2D5 = 6). There were no significant departures from the Hardy–Weinberg equilibrium for any of the markers across populations. All populations had similar percentages of heterozygosity and the effective allele number ranged from 2.00 to 2.92 (Table 1). Analysis of molecular variance revealed that 76 % of the variance was partitioned among individuals within populations (Table 3b). There was, however, significant (*P* < 0.001) and relatively high genetic differentiation among all populations (*φ*_PT_ = 0.243), among populations within regions (*φ*_PR_ = 0.133, *P* < 0.001) and between regions (*φ*_RT_ = 0.127, *P* < 0.001) (Table 3b). Pairwise *φ*_PT_ values between populations were variable, ranging from <0.001 to 0.427 (Table 4b). Non-hierarchical AMOVAs (not shown) revealed significant differentiation between Irish populations (*φ*_PT_ = 0.126; *P* < 0.001) and no differentiation between Spanish populations (*φ*_PT_ = 0.104; *P* = 0.065). In contrast to the AFLP results, the UPGMA grouped the two Spanish populations with the Irish Glencullen population. Within this group, the Glencullen population grouped most closely with the El Palancar population (Fig. 1B). No relationship was found between linear AFLP and SSR individual pairwise genetic distance matrices (*r*_xy_ = 0.360, *P* = 0.076).

### Floral width

**Figure 2.**
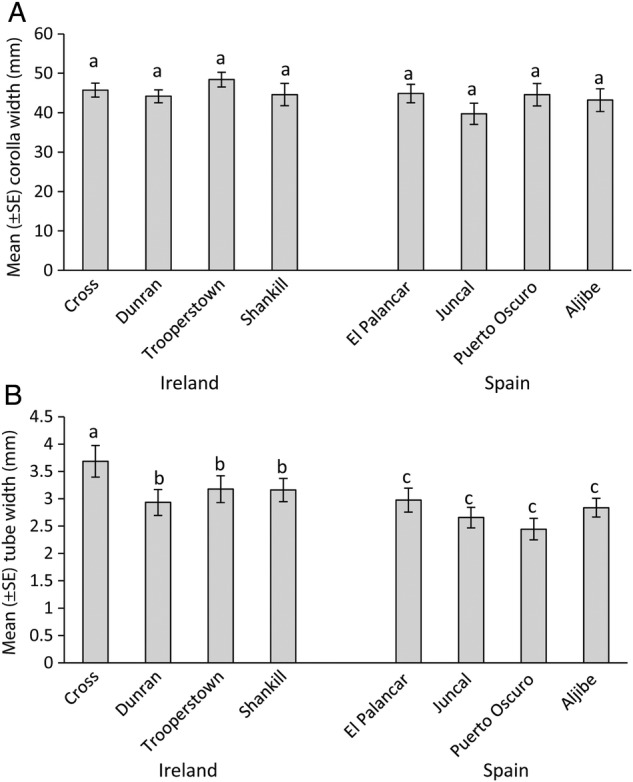
Mean (±SE) (A) corolla width in millimetre and (B) tube width in millimetre of flowers from each population within each region (Ireland and Spain). Means were calculated from five flowers from each of five plants within each population, and then for the population. Letters above scale bars correspond to the results of multiple comparison tests (SNK, *P*< 0.05).

There were no significant differences in corolla width between regions (mean ± SE: Ireland 45.72 ± 0.95, Spain 43.09 ± 1.18 mm) or between populations within regions (Table 5), but SNK tests revealed that there were significant differences among plants within populations in the Irish population at Shankill and in the Spanish populations (*P* < 0.05). There were significant differences in tube width between regions, among populations within regions and within populations (Table 5). Corolla tubes were significantly wider in Ireland compared with Spain (mean ± SE: Ireland 3.24 ± 0.16, Spain 2.73 ± 0.12 mm; Fig. 2). Student–Newman–Keuls post-hoc tests revealed that floral tubes were significantly wider in Crossover compared with the other populations in Ireland, and varied significantly between plants within populations in Ireland, but not in Spain (*P* < 0.05, Fig. 2B).

**Table 5.** Nested ANOVA results comparing corolla widths and tube widths of flowers between regions (Ireland and Spain), among populations (four per country, nested within regions) and within population (among five sampled plants nested within populations) (*n* = 5).

	Corolla width				Tube width			
	MS	*F*	Df	*P*	MS	*F*	Df	*P*
Between regions	346.37	3.02	1,6	0.133	13.00	6.76	1,6	0.041
Among populations	114.77	1.32	6,32	0.277	1.92	3.47	6,32	0.010
Within populations	86.94	4.93	32,160	<0.001	0.55	2.98	32,160	<0.001
Error	17.64				0.19

## Discussion

This study shows that both genetic diversity and floral width vary between populations of *R. ponticum*, but that native, rare populations are genetically and morphologically distinct from invasive populations. We found similar levels of genetic diversity in both declining native (Spanish) and invasive introduced (Irish) populations of *R. ponticum*. However, populations and regions were also genetically differentiated, a trend detected with both AFLP and SSR markers. Such genetic differentiation is expected when genetic drift and inbreeding occur in geographically isolated populations (Oakley and Winn 2012). Our findings of low genetic diversity and genetic similarity between Spanish and Irish populations support the findings of Erfmeier and Bruelheide (2011) using AFLP markers on native and invasive populations of *R. ponticum*. Although the level of diversity detected with AFLP and SSR markers was low, it was within the range for a species with a mixed mating system (Nybom and Bartish 2000). *Rhododenron ponticum* primarily relies on insect-mediated pollination for sexual reproduction (Mejías *et al.* 2002; Stout 2007*a*); however, clones may be prominent within populations resulting from vegetative spread (Mejías *et al.* 2002). We found 28 SSR genotypes in the Irish samples, of which 12 occurred more than once, and so may be clonal, with one to eight unique genotypes per population **[see Supporting Information]**. Therefore, pollen transfer between neighbouring plants may result in bi-parental inbreeding (where both parents are closely related), rather than outcrossing. In fact, observations of the behaviour of pollinators suggest that the levels of geitonogamy (pollen transfer among flowers of the same plant) may be high, since the main pollinators of *R. ponticum* in Ireland (*Bombus* spp.) tend to move between flowers on the same plant far more frequently than between flowers on different plants (Stout 2007*b*).

The native Spanish *R. ponticum* populations had more private AFLP bands (each population had 14 private bands) than invasive Irish ones (which had a mean of six private bands). Spanish populations contained fewer than 30 individual plants possibly due to range contraction and lack of sexual regeneration (Mejías *et al.* 2002). The introduced Irish populations were generally larger than the Spanish ones and were expanding as a result of both sexual and vegetative reproduction (Stout *et al.* 2006), but they may have derived from a small number of founding individuals. Thus Irish populations contained lower genetic variation and a lower number of private bands. Examination of polymorphic chloroplast DNA would be useful to explore founder effects further, as if there were a small number of founding individuals, we might expect low plastid diversity.

Our data show that native Spanish populations have probably experienced a recent genetic bottleneck as they have both low overall expected heterozygosity (*H*_j_), as estimated by AFLPs, and low allelic diversity, as estimated by SSRs. In addition, Irish Glencullen population groups with Spanish populations in both the AFLP and SSR analyses (and Irish Howth, Glencullen and Recess are not significantly differentiated from the Spanish Las Corzas population in AFLP pairwise *φ*_PT_ comparisons), which reveals the similarity of Irish and Spanish populations. However, the grouping of one of the Irish populations (Glencullen) with the Spanish populations could be due to homoplasy (a similar genetic structure due to convergence): this population also has more private alleles (alleles that are unique to a particular population from many populations sampled), and other Irish populations may have grouped together as they have fewer private alleles.

The population differentiation found, even between pairs of populations in each geographical location in Ireland (counties Galway, Kerry and Dublin), suggests that the gene flow is limited. This supports findings of Stephenson *et al.* (2007) who examined seed dispersal in this species and concluded that a very small proportion (0.02 %) of seeds moved >50 m, and Stout (2007*b*) who found that pollen dispersal was also likely to be limited (with 98 % of bee moves between flowers <1 m apart). Thus, with limited gene flow via both pollen and seeds, spread is likely to be the result of populations spreading in the form of an ‘invasion front’ and/or repeated introductions. Hence, management should focus on containing existing populations and preventing new introductions.

Corolla width varied much more within populations than between them, particularly in Spain. This suggests that this trait is not under strong selection pressure or is naturally highly variable, and our measurements are consistent with other descriptions of corolla width (e.g. Mejías *et al.* (2002) describe flowers as having a corolla of up to 6 cm in diameter). Tube width is clearly a highly variable trait in *R. ponticum*, varying between individuals within populations, among populations and between regions, similar to the patterns found for genetic diversity. No previous published studies have described tube width in *R. ponticum*. However, corolla tubes were, on average, >0.5 mm wider in Irish populations than in Spanish ones. Wider corolla tubes may be associated with increased pollinator visitation rates, because a greater range of pollinating insects can access the nectar from more open flowers with wider tubes. Wider corolla tubes in the introduced range may be an advantage, given that introduced species have to rely on native generalist insects for pollination. Indeed, a range of generalist visitor species were recorded visiting *R. ponticum* in Ireland, including solitary bees, bumblebees and hoverflies (Stout *et al.* 2006). The most common pollinators in Ireland are bumblebees, which have relatively long tongues (compared with hoverflies and solitary bees) and the ability to rob nectar if corolla tubes are too narrow for them to probe (Stout *et al.* 2006). However, the most effective pollinators are large queen bumblebees (Stout 2007*b*), which may be able to visit and collect nectar more efficiently if corolla tubes are wider (Suzuki 1994). Further comparisons of pollinator behaviour in the invasive and native range would be needed to test this hypothesis. Given that populations have diverged at a genetic level, we can also expect divergence at a morphological level (although the same populations were not compared for molecular and morphological analyses in this study). It may be non-adaptive processes, such as ecological sorting, different introduction times of *R. ponticum* to Ireland historically and phenotypic plasticity (i.e. changed phenotype in response to environmental conditions, e.g. Herrera 1992), that have a strong effect on the results observed. Indeed genetic drift and founder effects may explain the relatively low, though significant, levels of genetic differentiation observed. More work is needed to clarify whether such non-adaptive processes alone, or in combination with adaptive evolution in novel habitats, drive population differentiation in non-native populations. Although *R. ponticum* is very long-lived, it was introduced 200–250 years ago, and there has been time for post-introduction evolutionary change. Indeed, studies have found evidence for post-introduction selection affecting vegetative growth in *R. ponticum* (Erfmeier and Bruelheide 2005). Alternatively, initial introductions into Ireland may have been of wider tubed individuals; historical herbarium flower specimens could be used to investigate this, as long as the corolla tube is visible and intact. However, this is relatively unlikely given that there have been repeated introductions (Stephenson *et al.* 2006). In addition, there is little evidence for this floral difference to be a result of post-introduction hybridization with other *Rhododendron* species (Erfmeier *et al.* 2011).

## Conclusions

Using both the AFLP and SSR markers, we have shown that invasive *R. ponticum* in Ireland has low genetic diversity and populations are closely related to ancestral Spanish ones, but are also differentiated from one another, with limited between-population gene flow. Introduced individuals produce flowers with wider corolla tubes, which may attract more floral visitors due to increased nectar availability. The results of this study show that native Spanish populations are distinct and should be the focus of continuing conservation attention due to their restricted distribution and small size.

## Sources of Funding

This work was funded by an Enterprise Ireland Postdoctoral Fellowship (PD/2001/050), a Trinity Trust Award, a British Ecological Society Small Ecological Project Grant, a grant from the Percy Sladen Memorial Fund (Linnean Society) and a Science Foundation Ireland Research Frontiers Programme grant (10/RFP/EOB2842). K.J.D. is funded by the University of KwaZulu-Natal Postdoctoral fellowship.

## Contributions by the Authors

The lead author designed the study, obtained the funding, carried out fieldwork, laboratory work, data analysis and writing; all other authors contributed to study design, fieldwork, laboratory work, data analysis and writing.

## Conflicts of Interest Statement

None declared.

## Supporting Information

The following Supporting Information is available in the online version of this article –

**File S1.** 28 unique genotypes from 61 *R. ponticum* individuals in Irish populations identified by 4 SSR markers.

Additional Information
