# Supplementary material for: Genetic diversity and floral width variation in introduced and native populations of a long-lived woody perennial
Source: AoB Plants. 2014 Dec 19;7:plu087. doi: 10.1093/aobpla/plu087 (PMC4323518; doi:10.1093/aobpla/plu087)
Supplement: Additional Information [file supp_7_plu087_index.html]

Additional Information 

# Genetic diversity and floral width variation in introduced and native populations of a long-lived woody perennial

## Additional Information

Additional Information

**Files in this Data Supplement:**

- Supporting Information - pdf file
