# Supplementary material for: Genetic diversity and floral width variation in introduced and native populations of a long-lived woody perennial
Source: AoB Plants. 2014 Dec 19;7:plu087. doi: 10.1093/aobpla/plu087 (PMC4323518; doi:10.1093/aobpla/plu087)
Supplement: Additional Information [file supp_plu087_plu087supp.pdf]

Supporting Information. 28 unique genotypes from 61 *R. ponticum* individuals in Irish populations identified by 4 SSR markers.

Population	Genotype
Glencullen	A
Glencullen	A
Glencullen	A
Glencullen	C
Glencullen	C
Glencullen	C
Glencullen	D
Glencullen	H
Glencullen	I
Glencullen	O
Gortderraree	A
Gortderraree	C
Gortderraree	D
Gortderraree	E
Gortderraree	J
Gortderraree	K
Gortderraree	L
Gortderraree	W
Gortderraree	Z
Gortderraree	AB
Gortracussane	B
Gortracussane	B
Gortracussane	I
Gortracussane	I
Gortracussane	J
Gortracussane	K
Gortracussane	L
Gortracussane	N
Gortracussane	Q

Gortracussane	S
Gortracussane	X
Howth Head	A
Howth Head	B
Howth Head	B
Howth Head	B
Howth Head	B
Howth Head	B
Howth Head	K
Howth Head	M
Howth Head	R
Howth Head	AA
Kylemore	D
Kylemore	F
Kylemore	F
Kylemore	F
Kylemore	F
Kylemore	G
Kylemore	G
Kylemore	G
Kylemore	I
Kylemore	U
Recess	C
Recess	E
Recess	G
Recess	H
Recess	I
Recess	L
Recess	P
Recess	T
Recess	V
Recess	Y
